# The clinicopathological significance of miR-1307 in chemotherapy resistant epithelial ovarian cancer

**DOI:** 10.1186/s13048-015-0143-5

**Published:** 2015-04-09

**Authors:** Yingying Zhou, Min Wang, Jianlei Wu, Zhihui Jie, Shuang Chang, Ting Shuang

**Affiliations:** Department of Gynecology and Obstetrics, Shengjing Hospital of China Medical University, 36 Sanhao Street, Shenyang, Liaoning 110004 P.R. China

**Keywords:** miR-1307, Epithelial ovarian cancer, Chemotherapy resistant, MicroRNA, DAPK3

## Abstract

**Background:**

We aimed to examine the expression of miR-1307 in chemosensitive and chemoresistant epithelial ovarian cancer tissues and cell lines and to analyze the clinicopathological significance of miR-1307 in ovarian cancer.

**Methods:**

MicroRNA microarray was used to screen differentially expressed microRNAs between the chemosensitive and chemoresistant epithelial ovarian cancer tissues. RT-PCR was used to validate the candidate microRNA. The potential target genes and their enriched biological pathways of microRNA were also analyzed. Dual Luciferase Reporter Gene Assay was conducted to validate the regulation of miRNA-1307 on the 3’-UTR of DAPK3.

**Results:**

miRNA-1307 was up-regulated in the chemoresistant epithelial ovarian cancer tissues compared to the chemosensitive counterparts. The up-regulation of miRNA-1307 was not associated with menopause, tumor differentiation state, clinical stage, and lymph node metastasis of ovarian cancer. Gene ontology analysis of miR-1307 candidate target genes indicated that miR-1307 candidate target genes were enriched in the processes of cell proliferation and differentiation, nucleotide synthesis and metabolism, and lymphocytes activation.

**Conclusion:**

Our results suggest that miRNA-1307 may play a role in the development of chemoresistance in ovarian cancer.

**Electronic supplementary material:**

The online version of this article (doi:10.1186/s13048-015-0143-5) contains supplementary material, which is available to authorized users.

## Introduction

Epithelial ovarian carcinoma has the highest mortality rate in gynecological cancers. In fact, 52% of death caused by gynecological cancer are contributed by the epithelial ovarian carcinoma [1]. The current standard treatment strategy for epithelial ovarian carcinoma is cytoreductive surgery followed by platinum-based chemotherapy. Despite the advancements in the diagnosis and treatment of ovarian cancer, the 5-year survival rate for advanced ovarian cancer is poorly 30% [2].The main reasons for the high mortality of ovarian cancer include: early stage of ovarian cancer are usually asymptomatic; lack of diagnostic methods for early stage of ovarian cancer; and the development of chemoresistance during chemotherapy [3]. Past studies have shown that chemoresistance occurred in 1/3 of patients received platinum-based chemotherapy. In addition, nearly all recurrent ovarian tumors are resistant to platinum-containing chemotherapy drugs [4]. Because the development of chemoresistance is associated with a greatly reduced survival rate for ovarian cancer patients, there is an urgency to discover new therapeutic approaches to reduce the occurrence of chemoresistance.

MicroRNA (miRNA) is a class of small non-coding RNA molecule that was first discovered in 1993 [5]. miRNA plays an important regulatory role for gene expression by directly binding to the 3’ UTR of target mRNAs [6]. Numerous studies have demonstrated that miRNA is a critical factor in many biological processes, such as cell differentiation, cell proliferation, apoptosis, and energy metabolism [7]. Interestingly, miRNA has been shown to have dual roles in the development of cancer, either promotes carcinogenesis by inhibiting tumor suppressors or acts as tumor suppressors to down-regulate oncogenes [8]. Recently, there are increasing interests in studying the association between miRNA and ovarian cancer. Several studies have reported that miRNA is involved in the development of chemoresistance in ovarian cancer by inhibiting pro-apoptotic signal pathway [3]. The loss of let-7 has been considered contribute to the chemoresistance in ovarian cancer cells [9]. Up-regulation of miR-300 can inhibit cellular apoptosis via TGF-β signaling and thus promotes chemoresistance in ovarian cancer cells 11. Therefore, the dysregulation of miRNA may be a critical factor that determines the sensitivity of ovarian cancer cells to chemotherapy.

In this study, we conducted a miRNA screening using the miRNA microarray to search for differentially expressed miRNAs between the chemosensitive and chemoresistant ovarian cancer tissues. We then validated the results of miRNA microarray analysis.

## Materials and methods

### Ovarian tumor samples

8 cases of sporadic ovarian serous cystadenocarcinoma (4 chemoresistant and 4 chemosensitive) were used for the microRNA microarray analysis. All samples were obtained from surgical operations in the Shengjing hospital of the China Medical University. All cases were pathologically confirmed and no treatments were given before surgery. The age range for the chemoresistant group was from 42 to 79 years old with a median of 50.5. The age range for the chemosensitive group was from 33 to 71 years old with a median of 57.0. All patients received cytoreductive surgery and 6–8 cycles of TP chemotherapy regimen (paclitaxel + cisplatin) after surgery. Chest X-ray was obtained before surgery. Tumor response was evaluated in patient follow-up. Physical exam, ultrasound, and CA125 test were performed during each patient follow-up. CT, MRI and PET scan were also performed when it is necessary. NCCN guideline for ovarian cancer was used to determine the chemosensitivity and resistance. A tumor is chemosensitive if there is no tumor recurrence within 6 months of the initial chemotherapy and chemoresistant if there is. The study was approved by the Ethic Committee of Shengjing Hospital of China Medical University, approval number was 2014PS14K. All patients provided informed consents.

40 cases of ovarian serous cystadenocarcinoma (20 chemoresistant and 20 chemosensitive) were used in the RT-PCR validation for candidate miRNA obtained from the miRNA microarray screening. All samples were obtained from surgically dissected tumors in the Shengjing hospital of the China Medical University between January 2009 and January 2012. Patients received the TP chemotherapy regimen (paclitaxel + cisplatin) after surgery. Tumor response was evaluated in patient follow-up for 6 months. The criteria for chemosensitivity and resistance is the same as above. Clinical staging were based on the International Federation of Gynecology and Obstetrics (FIGO) stage.

### Cell culture

Human ovarian cancer cell line SKOV3 and SKOV3-TR30 (resistant to paclitaxel) was obtained from the Women’s hospital of the Zhejiang University School of Medicine. SKOV3 cells were maintained in RPMI-1640 medium (10% FBS, 100 μg/ml penicillin and 100 μg/ml streptomycin). SKOV3-TR30 cells were maintained in the same medium as SKOV3 with 30 nmol/L paclitaxel. All cells were incubated at 37°C with 5% CO2.

### MicroRNA microarray

Affymetrix microRNA microarray was used in this study (GeneChip® miRNA 4.0 Array, Affymetrix). Experiment was performed according to the instruction of the manufacturer. The Cluster 3.0 software was used for the analysis of microRNA expression. The criteria for differentially expressed miRNA is |Score (d)| ≥2, and fold change ≥2 or ≤0.5.

### RT-PCR

Total RNA were extracted from 50-100 mg of frozen tumor tissues using Trizol (Invitrogen). Reverse transcription was performed according to manufacturer’s instruction (Primescript TM RT reagent Kit, TaKaRa). The TagMan microRNA kit (TaKaRa) was used for the real time PCR to assess the expression level of the candidate microRNA. PCR cycle conditions are: 95°C for 5 minutes, followed by 40 cycles of 95°C 10s, 60°C 20s, 72°C 20s, and 78°C 20s. The primers for miR-1307 are: forward primer 5’-AACTCGGCGTGGC -3’; reverse primer 5’-GAGCAGGCTGGAGAA-3’. U6 was used as internal control in RT-PCR: forward primer 5’-GCTTCGGCAGCACATATACTAAAAT-3’; reverse primer 5’-CGCTTCACGAATTTGCGTGTCAT-3’; Experiment was performed independently for three times.

### Analysis of miRNA target genes and gene ontology analysis

TargetScan, miRanda and Diana microT-CDS were used to analyze the potential target genes for miR-1307. GOstat was used for gene enrichment analysis. The DAVID database was used for signal transduction enrichment analysis.

### Reporter assay for DAPK3

pMIR-REPORT-DAPK3 and pMIR-REPORT-DAPK3-mut reporters were used to validate the regulatory effect of miRNA-1307 on DAPK3. The 336 bps of DAPK3 (NM_001348) contains a potential miRNA-1307 binding site in the 3’-UTR were cloned into the pMIR-REPORT vector by the SpeI and HindIII restriction enzyme cutting sites. The site-directed mutagenesis kit (Beyotime, China) was used to induce five nucleotides mutations in the pMIR-REPORT-DAPK3-mut. The mutation on DAPK3 was confirmed by DNA sequencing (from CGCCGAG to CATTCTG). For luciferase reporter assay, HEK293 cells were seeded in 24-well plates for 24 hours to reach confluency of 70-80% before transfecting reporter constructs. pMIR-REPORT-DAPK3 and pMIR-REPORT-DAPK3-mut were then transfected into HEK293 cells with Renilla luciferase normalization plasmid (pELTK). Luciferase reporter activity was measured 24 hours after transfection and normalized by Renilla reporter signals (Promega).

### Statistical analysis

The Fisher Exact Test was used to calculate *p* value. ANOVA was used to compare the mean values of the results of luciferase assay among the three groups. The Bonferroni test was used to compare the mean values of the luciferase assay results between two groups. A *p* value <0.05 indicated statistically significant. SPSS17.0 was used for statistical analysis.

## Results

### Upregulation of miR-1307 in chemoresistant ovarian tumors

**Figure 1:**
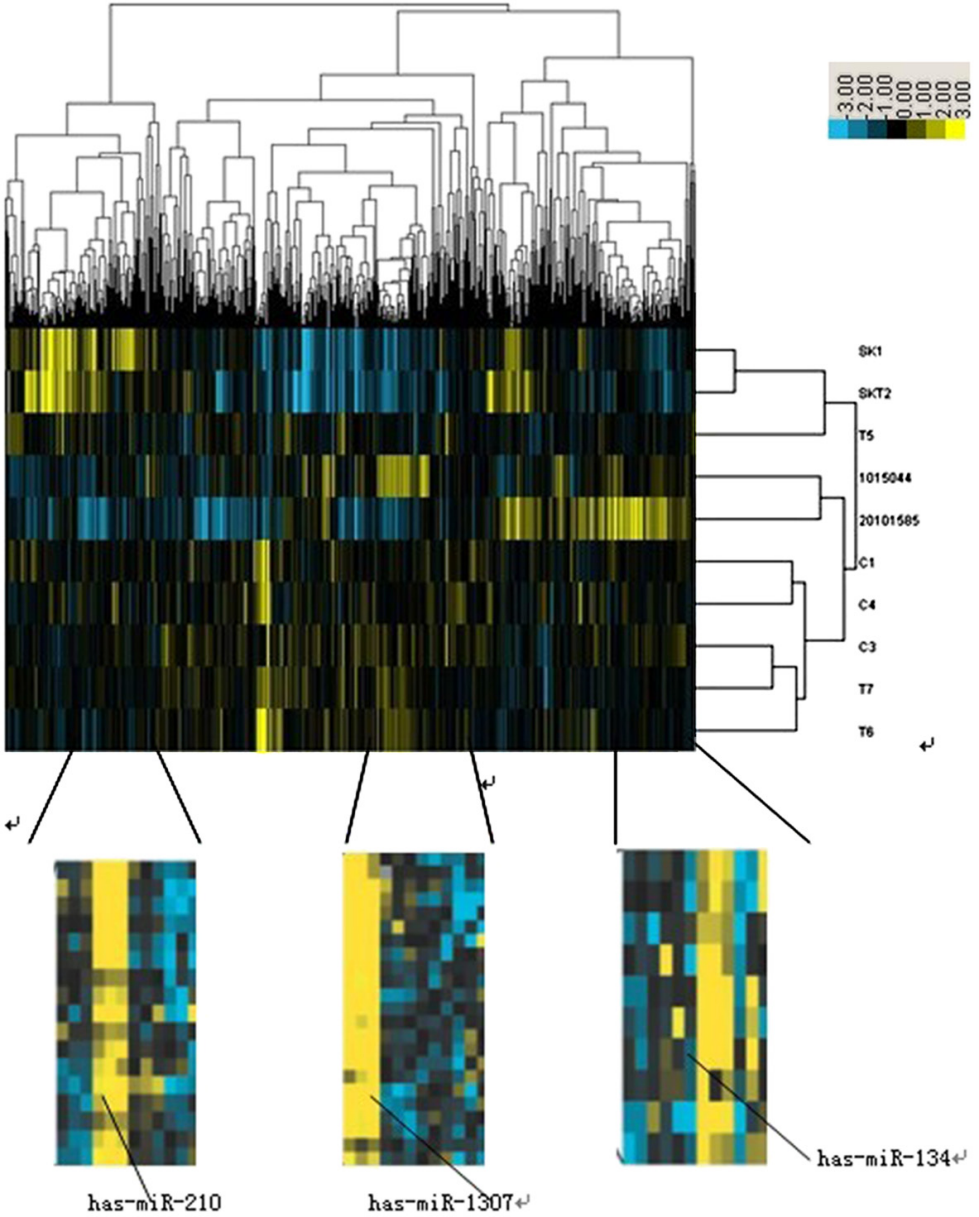
**MicroRNA microarray screening for differentially expressed miRNAs.** C1, C3, C4 and 20101585 were chemosensitive ovarian tumors, while T5, T7, T8 and 1015044 were chemoresistant ovarian cancer tissues. SK1 and SKT2 were chemosensitive and chemoresistant ovarian cancer cell lines respectively. The miRNA expression profile of miR-210, miR-1307 and miR-134 were also shown.

**Figure 2:**
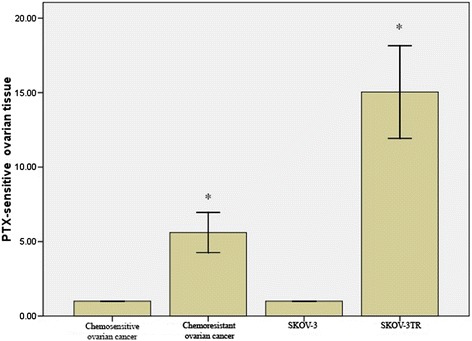
**Upregulation of miR-1307 in chemoresistant ovarian cancer tissues and cell line.** RT-PCR analysis of miR-1307 expression in 20 chemosensitive ovarian cancer tissues and 20 chemoresistant ovarian cancer tissues were compared. The relative expression level of chemosensitive SKOV-3 and chemoresistant SKOV-3TR cells were also shown. *indicates *p* = 0.001.

To search for differentially expressed miRNA that may play a role in the development of chemoresistance in ovarian cancer, we compared the miRNA expression profile between 4 chemosensitive and 4 chemoresistant ovarian tumor samples. As a result, we discovered that miR-1307 was upregulated in chemoresistant ovarian tumors when compared to the chemosensitive ovarian tumors (Figure 1). To validate this result, we performed quantitative RT-PCR for miR-1307 in 20 chemosensitive and 20 chemoresistant ovarian carcinoma samples. We found that the expression level of miR-1307 was 4.41 folds higher in chemoresistant ovarian tumors compared to the chemosensitive ovarian tumors (*p* = 0.001, Figure 2). Consistently, the expression level of miR-1307 was 2.81 folds higher in the SKOV3-TR30 cells, which is a chemoresistant clone of the ovarian cancer cell line SKOV3, than the chemosensitive SKOV3 cells (*p* = 0.001, Figure 2).

### Cliniopathological relevance of miR-1307 in ovarian cancer

To examine the clinicopathological relevance of miR-1307 upregulation in ovarian cancer, we analyzed the level of miR-1307 with a number of clinical and pathological parameters. We found that there was no statistically significant association between the expression level of miR-1307 and menopause, tumor differentiation status, clinical staging and lymph node involvement (Table 1). To determine the cutoff value of miR-1307 as a predicting factor for chemoresistance in ovarian cancer, we performed the ROC analysis. We thus found the cut-of level was 2.1579, which suggested that chemoresistance of paclitaxel may be developed if 2^-△△Ct^ of miR-1307 is more than 2.1579 in RT-PCR and change of therapeutic regimen should be considered.

**Table 1:** C**linicopathological relevance of miR-1307 upregulation in ovarian carcinoma**

**Parameters**	**Case no.**	**miR-1307 level**	***p*** **value**
**Age**			0.8011
Postmenopause	25	2.137 ± 1.1457	
Before menopause	15	2.540 ± 1.1282	
**Differentiation status**			0.6080
High or medium	18	2.917 ± 1.0120	
Low	22	2.138 ± 1.297	
**Clinical stage**			0.4985
Stage I	3	1.404 ± 0.8790	
Stage II	8	3.880 ± 1.4341	
Stage III	29	3.945 ± 1.3631	
**Lymph node metastasis**			0.3257
Yes	15	2.497 ± 1.3521	
No	25	4.347 ± 2.0142	

### Analysis of miR-1307 candidate target genes

To gain an insight to the regulatory network of miR-1307, we performed bioinformatics analysis to identify potential miR-1307 target genes. As a result, an analysis of Targetscan、miRanda、Pita returned 124 common candidate genes for miR-1307. Biological pathway analysis showed that miR-1307 candidate genes were enriched in organogenesis, gene transcription, cell proliferation and differentiation, neurogenesis, T cell activation, vitamin synthesis and metabolism pathways (Additional file 1).

**Figure 3:**
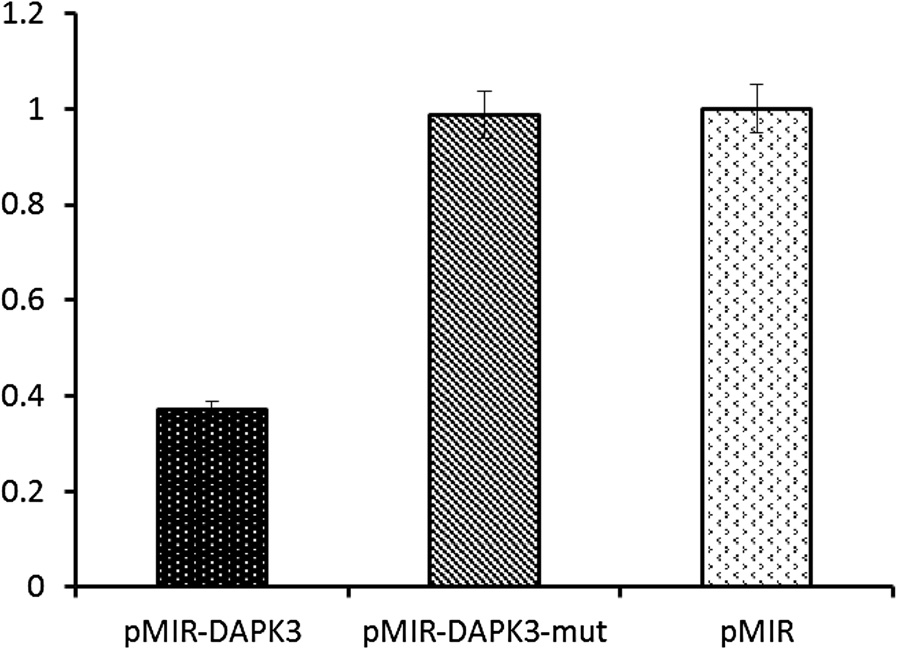
**Validation of DAPK3 as a target of miRNA-1307.** Luciferase reporter assay using pMIR-REPORT-DAPK3, pMIR-REPORT-DAPK3-mut and control were shown.

To validate the targets of miRNA-1307, we constructed the reporter assay system for DAPK3, which is a potential regulatory target of miRNA1307 that has been consistently identified in our bioinformatics analysis. We found that the reporter activity level of DAPK3 was significantly reduced compared to the control (p = 0.01, Figure 3). This result was confirmed when the suppressive effect of miRNA-1307 was abolished on the mutated DAPK3 reporter.

## Discussion

miRNA has been long linked to the development of chemoresistance [10]. With the advancement of miRNA microarray technology, we can now evaluate the miRNA expression profile in a relatively short period of time. In this study, we used the miRNA microarray to search for differentially expressed miRNAs that are specific to chemoresistance in epithelial ovarian carcinoma. As a result, we found that miR-1307 was upregulated in chemoresistant ovarian cancer when compared to the chemosensitive ovarian cancer. This result was confirmed by RT-PCR in 20 chemoresistant and 20 chemosensitive ovarian cancer tissues, indicating that miR-1307 was associated with chemoresistance in ovarian cancer.

microRNA is an important post-transcriptional regulator of gene expression. miRNA has been estimated to regulate more than 30% of mRNAs in human cells [11]. Studies have demonstrated that multiple miRNAs can regulate one gene while one miRNA can regulate multiple target genes, weaving a complex regulatory network for the delicate regulation of gene expression in eukaryotic cells [12]. In the past decade, numerous studies have shown that dysregulation of miRNA may contribute to the development of cancer. For examples, miR-15a, miR-16-1, and miR-126 act as tumor suppressor to downregulate oncogenes [13,14]. In contrast, miR-135, MiR-522, miR-15b, and miR-490-3p function to promote cancer development and progression [15-18]. In addition, miRNA has been shown to regulate noncoding regions of genome that are important in the tumorigenesis [19].

Recently, many research have shown that the resistance of ovarian cancer to chemotherapeutic drugs is regulated by miRNA. There is a significant difference in miRNA expression profile between the chemosensitive and chemoresistant ovarian cancer cells. The study by Yu et al. showed that the downregulation of miR-29 increased resistance to cisplatin in ovarian cancer cells [20]. MiR-106a and miR-591 have important roles in conferring paclitaxel resistance to ovarian cancer cells, and modulating miRNAs to resensitize paclitaxel-resistant cancer cells by regulating BCL10, caspase-7, and ZEB1 [21]. In the study by Cittelly et al., the chemoresistant ovarian cancer cells became chemosensitive when miR-200c was ectopically expressed [22]. In another study, Prislei et al. found that high level of miR-200c inhibited the expression of TUBB3 and was associated with a better prognosis in ovarian cancer [23]. Moreover, miRNA-182, miR-376c, and miR-141 have been shown to promote ovarian cancer growth, metastasis and chemoresistance by regulating signaling pathways function in these processes [24-26]. Interestingly, studies have shown that miRNA-152 and miRNA-185 can function synergistically to increase the chemosensitivity of ovarian cancer cells to the platinum-based chemotherapy [27].

miR-1307 is located at the intron region of USMG5 gene in chromosome 10. Has-miR-1307-3p is originated from the 3’ end of pre-miR-1307 while has-miR-1307-5p comes from the 5’ end of pre-miR-1307. The function of miR-1307 is still largely unknown. Zhu et al. reported that 3 miRNAs (hsa-miR-1301, hsa-miR-1307, and hsa-miR-2110) that were specific to the Epstein-Barr virus-induced oropharyngeal cancer [28]. However, the role of miR-1307 in the pathogenesis of Epstein-Barr virus-induced oropharyngeal cancer has not been studied. Morin et al. examined small molecular RNA in embryonic stem cells and embryoid body by massive parallel signature sequencing [29]. They discovered that the level of miR-1307 was significantly higher in embryonic stem cells than the more differentiated embryoid body, suggesting that miR-1307 plays a role in the regulation of embryonic stem cell self-maintenance and differentiation. Although the functional studies of miR-1307 are still very limited, current evidences support that miR-1307 is associated with cell proliferation, differentiation, and possibly tumorigenesis. Consistently, our results indicated that miR-1307 was upregulated in chemoresistant ovarian cancer cell lines and tumor tissues, implying an association between miR-1307 and chemoresistance in ovarian cancer. However, the exact role of miR-1307 in the development of chemoresistance need to be elucidated in future studies.

In the clinicopathological analysis of miR-1307 and epithelial ovarian cancer, we did not find significant associations between the expression level of miR-1307 and the menopause, tumor differentiation status, clinical stages and lymph node involvement, suggesting that miR-1307 may not play a role in the ovarian cancer growth and progression but specific to the development of chemoresistance. Hence, miR-1307 may be used as a biomarker of chemosensitivity for ovarian cancer patients received the platinum-based chemotherapy.

In the bioinformatics analysis, we identified 124 potential candidate targets of miR-1307, which were enriched in many biological processes that are important in tumorigenesis, such as the nucleotide synthesis and metabolism, cell proliferation and metabolism. Since the role of miR-1307 in the development of chemoresistance is still largely unknown, the study of regulatory targets of miR-1307 may shed light on the function of miR-1307.

In this study, we successfully validated DAPK3 as a target of miRNA-1307 using the reporter assay. DAPK3 has been consistently identified in our analysis for the potential target of miRNA-1307. Our analysis showed that the 3’-UTR of DAPK3 contains potential miRNA-1307 binding sites. DAPK, is also called zipper-interacting protein kinase or DAPK-like kinase, is located on 19q13.3. The product of DAPK is a nuclear protein with serine/threonine kinase activity. The C-terminal of DAPK3 contains a leucine zipper motif. The N-terminal of DAPK3 includes a pro-apoptotic protein kinase domain, which is a shared by all members of the DAPK family. DAPK3 has been show to function in apoptosis, autophagy, actin filament regulation, cell migration, smooth muscle contraction, mitosis, and development of several types of cancer [30,31]. Since DAPK3 induced cell apoptosis is mediated by its intrinsic kinase activity, mutations in the kinase domain of DAPK3 could promote cell survival, proliferation, and development of chemoresistance [32]. Previous studies have shown that DAPK3 is widely expressed in many tissues and having the highest expression level in female reproductive system such as uterus, ovary, and placenta [33]. The frequency of DAPK3 kinase domain mutations in lung, ovary, and colorectal cancer is 3.2% compared to the frequency of 1.4% in all types of cancer, while no DAPK3 mutations have been observed in the corresponding normal tissues [34]. Past studies have shown that the mutations in DAPK3 kinase domain increased the anti-apoptotic and survival abilities of tumor cells and thus reduced the chemosensitivity of tumor cells [35,36]. However, DAPK3 mutations have not been detected in all cancer samples. The expression level of DAPK3 have been shown to regulate the survival and apoptosis in tumor cells. Overexpression of DAPK3 induced changes in tumor cell morphology, suppressed cell aggregation, and promoted cell apoptosis [32,37]. Therefore, the expression level of DAPK3 could be a marker for cancer migration, invasion, and survival. Overall, the mutations and altered expression of DAPK3 are associated with the proliferation of cancer cells and the development of chemoresistance. It will be very interesting to know if DAPK3 has a role in the miRNA-1307 associated chemoresistance in ovarian cancer.

The development of chemoresistance is a complex process that involved the regulation of multiple factors, including miRNA. In this study, we discovered that miR-1307 was upregulated in chemoresistant ovarian cancer cell lines and tumor tissues compared to the chemosensitive counterparts, supports that miR-1307 is associated with the development of chemoresistance in ovarian cancer. Because the functional study of miR-1307 is still very limited and the bioinformatics analysis are mostly based on theoretical prediction, how miR-1307 contributes to the chemoresistance in ovarian cancer through the regulation of target genes is still unclear. Therefore, future study is needed to clarify the function of miR-1307 in chemoresistance and to provide theoretical basis for the development of new therapeutics to target miR-1307. Our results demonstrated that miRNA-1307 is upregulated in chemoresistant ovarian cancer tissues compared to the chemosensitive counterparts, providing novel evidences to support that miR-1307 may have a role in the development of chemoresistance in ovarian cancer cells.

## Additional file

Additional file 1:
**GO analysis of miR-1307 candidate target genes.** Biological pathway analysis showed that miR-1307 candidate genes were enriched in organogenesis, gene transcription, cell proliferation and differentiation, neurogenesis, T cell activation, vitamin synthesis and metabolism pathways.
