# Supplementary material for: The clinicopathological significance of miR-1307 in chemotherapy resistant epithelial ovarian cancer
Source: J Ovarian Res. 2015 Apr 9;8:23. doi: 10.1186/s13048-015-0143-5 (PMC4449560; doi:10.1186/s13048-015-0143-5)
Supplement: Additional file 1: — GO analysis of miR-1307 candidate target genes. Biological pathway analysis showed that miR-1307 candidate genes were enriched in organogenesis, gene transcription, cell proliferation and differentiation, neurogenesis, T cell activation, vitamin synthesis and metabolism pathways. [file 13048_2015_143_MOESM1_ESM.doc]

| Number_symbols_within_mylist_and_GO | Number_symbols_within_reflist_and_GO | Number_symbols_within_mylist | Number_symbols_within_reflist | Symbols_within_mylist | Enrichment_Score | Pvalue | Qvalue |
| --- | --- | --- | --- | --- | --- | --- | --- |
| 18 | 1090 | 102 | 15507 | ABLIM2,AP2A2,CACNA1C,CLASP1,DAPK3,  GNAO1,IGF1R,ITGA3,LBX1,LHX5,MAPK8IP3,  NRBP2,PAX2,PIP5K1C,SOX8,SS18L1,  TLX2,TLX3 | 2.510577442 | 0.000244 | 0.182247 |
| 2 | 4 | 102 | 15507 | PAX2,SOX8 | 76.01470588 | 0.000255 | 0.182247 |
| 2 | 4 | 102 | 15507 | PAX2,SOX8 | 76.01470588 | 0.000255 | 0.182247 |
| 17 | 1007 | 102 | 15507 | ABLIM2,AP2A2,CACNA1C,CLASP1,DAPK3,  GNAO1,IGF1R,LBX1,LHX5,MAPK8IP3,NRBP2,  PAX2,PIP5K1C,SOX8,SS18L1,TLX2,TLX3 | 2.56653426 | 0.000288 | 0.182247 |
| 24 | 1749 | 102 | 15507 | ABLIM2,AP2A2,CACNA1C,CLASP1,DAPK3,  GNAO1,IGF1R,ITGA3,LBX1,LHX5,LRCH4,MAPK8IP3,  NRBP2,NRGN,PAX2,PDX1,PIP5K1C,PITX1,SCN5A,  SOX8,SS18L1,TAGLN3,TLX2,TLX3 | 2.086166885 | 0.000336 | 0.182247 |
| 18 | 1158 | 102 | 15507 | ABLIM2,AP2A2,CACNA1C,CLASP1,DAPK3,GNAO1,  IGF1R,ITGA3,LBX1,LHX5,MAPK8IP3,NRBP2,PAX2,PIP5K1C,SOX8,SS18L1,TLX2,TLX3 | 2.363151478 | 0.000508 | 0.182247 |
| 6 | 169 | 102 | 15507 | CACNA1C,CHRNA4,CHST10,ITGA3,PRKCZ,SLC12A5 | 5.397493909 | 0.000871 | 0.182247 |
| 14 | 835 | 102 | 15507 | ANKRD11,CARM1,FOXQ1,HEG1,HOXC11,IGF1R,LBX1,MAPK8IP3,PAX2,PDX1,ROR2,SCN5A,SOX8,STAT5A | 2.548996125 | 0.001111 | 0.182247 |
| 2 | 8 | 102 | 15507 | IGF1R,PDX1 | 38.00735294 | 0.001169 | 0.182247 |
| 2 | 8 | 102 | 15507 | PAX2,SOX8 | 38.00735294 | 0.001169 | 0.182247 |
| 2 | 8 | 102 | 15507 | PAX2,SOX8 | 38.00735294 | 0.001169 | 0.182247 |
| 5 | 127 | 102 | 15507 | AGPAT6,ANKRD11,HEG1,SLC12A5,STAT5A | 5.985409912 | 0.001504 | 0.182247 |
| 36 | 3451 | 102 | 15507 | ABLIM2,AGPAT6,ANKRD11,AP2A2,CACNA1C,CARM1,CLASP1,DAPK3,FOXQ1,GATA5,GNAO1,HEG1,HOXC11,IGF1R,ITGA3,LBX1,LHX5,LRCH4,MAPK8IP3,NRBP2,NRGN,PAX2,PDX1,PIP5K1C,PITX1,PRKCZ,ROR2,SCN5A,SIN3B,SOX8,SS18L1,STAT5A,TAGLN3,TLX2,TLX3,ZBTB32 | 1.585934171 | 0.00177 | 0.182247 |
| 8 | 346 | 102 | 15507 | CACNA1C,CHRNA4,CHST10,GNAO1,GNG7,ITGA3,PRKCZ,SLC12A5 | 3.515130908 | 0.002013 | 0.182247 |
| 40 | 4012 | 102 | 15507 | ABLIM2,AGPAT6,ANKRD11,AP2A2,CACNA1C,CARM1,CLASP1,DAPK3,DGKD,FOXQ1,GATA5,GNAO1,HEG1,HOXC11,IGF1R,INTS1,ITGA3,LBH,LBX1,LHX5,LRCH4,MAPK8IP3,NRBP2,NRGN,PAX2,PDX1,PIP5K1C,PITX1,PRKCZ,PRM2,ROR2,SCN5A,SIN3B,SOX8,SS18L1,STAT5A,TAGLN3,TLX2,TLX3,ZBTB32 | 1.515746877 | 0.00209 | 0.182247 |
| 88 | 11490 | 102 | 15507 | ABLIM2,AGPAT6,AKT1S1,ANKRD11,AP2A2,BAIAP3,BRD3,C21orf2,CABP1,CACNA1C,CAPS,CARM1,CHFR,CHRNA4,CHST10,CLASP1,CYP8B1,DAPK3,DGKD,DNMBP,FOXQ1,FTSJ2,GATA5,GMDS,GNAO1,GNG7,GPR68,HEG1,HOXC11,IGF1R,IL2RB,INTS1,ITGA3,KCNJ12,KCNT1,LBH,LBX1,LHX5,LRCH4,MAP3K14,MAPK8IP3,MVD,MXD4,NCF1,NRBP2,NRGN,NUP62,OR2C3,PANX2,PAX2,PDIA4,PDX1,PDXK,PIP5K1C,PITX1,PPM1F,PPP2R4,PRKCZ,PRM2,RAB35,RASSF2,RIMS4,ROR2,RRBP1,SCN5A,SIN3B,SLC12A5,SLC24A6,SLC29A4,SOX8,SS18L1,STAT5A,SURF4,TAGLN3,TIMM13,TLX2,TLX3,TNIP1,TNPO2,TNRC6B,TOR1B,TRAF7,UNC93B1,UPF1,VPS37C,VPS4B,WIPI2,ZBTB32 | 1.164367993 | 0.002148 | 0.182247 |
| 16 | 1109 | 102 | 15507 | ABLIM2,BRF1,FOXQ1,GATA5,LBH,LHX5,NUP62,PAX2,PDX1,PITX1,ROR2,SOX8,SS18L1,STAT5A,TNIP1,TNRC6B | 2.193390972 | 0.002324 | 0.182247 |
| 12 | 708 | 102 | 15507 | GNAO1,IGF1R,LBX1,LHX5,MAPK8IP3,PAX2,PDX1,PITX1,SCN5A,SOX8,TAGLN3,TLX3 | 2.576769691 | 0.00234 | 0.182247 |
| 2 | 12 | 102 | 15507 | AGPAT6,DGKD | 25.33823529 | 0.002709 | 0.182247 |
| 2 | 12 | 102 | 15507 | AGPAT3,AGPAT6 | 25.33823529 | 0.002709 | 0.182247 |
| 17 | 1237 | 102 | 15507 | ABLIM2,BRF1,FOXQ1,GATA5,IGF1R,LBH,LHX5,NUP62,PAX2,PDX1,PITX1,ROR2,SOX8,SS18L1,STAT5A,TNIP1,TNRC6B | 2.089329022 | 0.002803 | 0.182247 |
| 16 | 1140 | 102 | 15507 | AP2A2,BAIAP3,CACNA1C,CHRNA4,GNG7,HOXC11,KCNJ12,LHX5,PANX2,PDX1,PIP5K1C,PRKCZ,SCN5A,SLC12A5,SLC24A6,SOX8 | 2.13374613 | 0.003061 | 0.182247 |
| 5 | 150 | 102 | 15507 | CARM1,HOXC11,PAX2,ROR2,SOX8 | 5.067647059 | 0.00311 | 0.182247 |
| 16 | 1143 | 102 | 15507 | ABLIM2,BRF1,FOXQ1,GATA5,LBH,LHX5,NUP62,PAX2,PDX1,PITX1,PPM1F,ROR2,SOX8,SS18L1,STAT5A,TNIP1 | 2.128145746 | 0.003142 | 0.182247 |
| 2 | 13 | 102 | 15507 | AGPAT3,AGPAT6 | 23.38914027 | 0.003188 | 0.182247 |
| 23 | 1945 | 102 | 15507 | ABLIM2,BRF1,FOXQ1,GATA5,IGF1R,LBH,LHX5,MAP3K14,MAPK8IP3,NUP62,PAX2,PDX1,PITX1,PPM1F,PPP2R4,PRKCZ,ROR2,SOX8,SS18L1,STAT5A,TNIP1,TNRC6B,TRAF7 | 1.797777106 | 0.003413 | 0.182247 |
| 17 | 1262 | 102 | 15507 | ABLIM2,BRF1,FOXQ1,GATA5,IGF1R,LBH,LHX5,NUP62,PAX2,PDX1,PITX1,ROR2,SOX8,SS18L1,STAT5A,TNIP1,TNRC6B | 2.047939778 | 0.003446 | 0.182247 |
| 39 | 3982 | 102 | 15507 | ABLIM2,AGPAT6,ANKRD11,AP2A2,C21orf2,CACNA1C,CARM1,CLASP1,DAPK3,FOXQ1,GATA5,GNAO1,HEG1,HOXC11,IGF1R,INTS1,ITGA3,LBX1,LHX5,LRCH4,MAPK8IP3,NRBP2,NRGN,PAX2,PDX1,PIP5K1C,PITX1,PRKCZ,PRM2,ROR2,SCN5A,SIN3B,SOX8,SS18L1,STAT5A,TAGLN3,TLX2,TLX3,ZBTB32 | 1.488987207 | 0.003457 | 0.182247 |
| 5 | 156 | 102 | 15507 | CACNA1C,CHST10,ITGA3,PRKCZ,SLC12A5 | 4.872737557 | 0.003678 | 0.182247 |
| 2 | 14 | 102 | 15507 | PAX2,SOX8 | 21.71848739 | 0.003703 | 0.182247 |
| 2 | 14 | 102 | 15507 | PAX2,SOX8 | 21.71848739 | 0.003703 | 0.182247 |
| 15 | 1056 | 102 | 15507 | ABLIM2,BRF1,FOXQ1,GATA5,LBH,LHX5,NUP62,  PAX2,PDX1,PITX1,ROR2,SOX8,SS18L1,STAT5A,TNIP1 | 2.15950869 | 0.003717 | 0.182247 |
| 6 | 229 | 102 | 15507 | HOXC11,LBX1,PAX2,PITX1,SOX8,TLX3 | 3.983303365 | 0.004028 | 0.182247 |
| 2 | 15 | 102 | 15507 | LBX1,LHX5 | 20.27058824 | 0.004254 | 0.182247 |
| 22 | 1869 | 102 | 15507 | AP2A2,BAIAP3,CABP1,CACNA1C,CHRNA4,CLASP1,NCF1,NUP62,PDIA4,PDX1,PIP5K1C,PRKCZ,RAB35,RIMS4,  SLC24A6,SURF4,TIMM13,TNPO2,UNC93B1,UPF1,VPS37C,VPS4B | 1.789538287 | 0.004491 | 0.182247 |
| 20 | 1651 | 102 | 15507 | ABLIM2,ANKRD11,AP2A2,C21orf2,CACNA1C,CARM1,CLASP1,FOXQ1,HEG1,HOXC11,IGF1R,LBX1,LHX5,MAPK8IP3,PAX2,PIP5K1C,ROR2,SOX8,STAT5A,TLX2 | 1.841664588 | 0.005009 | 0.182247 |
| 29 | 2753 | 102 | 15507 | ABLIM2,AGPAT6,AP2A2,CACNA1C,CARM1,CLASP1,DAPK3,GATA5,GNAO1,HEG1,HOXC11,IGF1R,ITGA3,LBX1,LHX5,MAPK8IP3,NRBP2,PAX2,PDX1,PIP5K1C,PITX1,PRKCZ,PRM2,ROR2,SOX8,SS18L1,STAT5A,TLX2,TLX3 | 1.601472191 | 0.005148 | 0.182247 |
| 3 | 54 | 102 | 15507 | LBX1,SOX8,TLX3 | 8.446078431 | 0.005375 | 0.182247 |
| 2 | 17 | 102 | 15507 | CHRNA4,PRKCZ | 17.88581315 | 0.005463 | 0.182247 |
| 3 | 56 | 102 | 15507 | H6PD,NMNAT1,PDXK | 8.144432773 | 0.00595 | 0.182247 |
| 2 | 18 | 102 | 15507 | PAX2,SOX8 | 16.89215686 | 0.00612 | 0.182247 |
| 2 | 18 | 102 | 15507 | PAX2,SOX8 | 16.89215686 | 0.00612 | 0.182247 |
| 2 | 18 | 102 | 15507 | PAX2,SOX8 | 16.89215686 | 0.00612 | 0.182247 |
| 9 | 508 | 102 | 15507 | FOXQ1,IGF1R,LBX1,PAX2,PDX1,ROR2,SOX8,STAT5A,TLX2 | 2.69343446 | 0.006258 | 0.182247 |
| 1 | 1 | 102 | 15507 | PAX2 | 152.0294118 | 0.006578 | 0.182247 |

Red colored gene are genes identified in both GO analysis and DAVID gene analysis
